# Youth engagement in global conservation governance

**DOI:** 10.1111/cobi.14387

**Published:** 2024-11-25

**Authors:** Samantha S. Sithole, Gretchen M. Walters, Philile Mbatha, Frank Matose

**Affiliations:** ^1^ Institute of Geography and Sustainability University of Lausanne Lausanne Switzerland; ^2^ Environmental and Geographical Science Department University of Cape Town Cape Town South Africa; ^3^ People, Rights and Resources in Africa Sociology Department University of Cape Town Cape Town South Africa

**Keywords:** conservation, environmental justice, global environmental governance, youth, youth engagement, conservación, gestión ambiental, justicia ambiental, juventud, participación juvenil, 青年, 青年参与, 全球环境治理, 环境正义, 保护

## Abstract

Youth are increasingly recognized for their important role in shaping environmental decisions surrounding conservation. Regrettably, youth who are crucial decision‐makers are often excluded from environmental governance spaces due to structural barriers, both economic and political. As highlighted by recent environmental justice literature, this marginalization hinders their active participation in the decision‐making process. The recent publication of the International Union for Conservation of Nature (IUCN) Youth Strategy 2022–2030 has brought prominent environmental organizations into the debate. The IUCN World Conservation Congress (WCC) presents a useful example from which to understand how youth access and participate in decision‐making at the highest level of governance in a prominent global conservation organization. We used event ethnography and participant observation methods to study the WCC Forum in Marseille, France (2021). We sought to examine the geopolitical intricacies of power and the underlying inequalities at the root of youth engagement, or lack thereof. We considered the IUCN's engagement with youth, outlining the process from previous resolutions and recommendations to the publication of the IUCN Youth Strategy in 2022. The results from the youth narratives we compiled showed that youth are not a monolith, that tokenism should be challenged, and that youth have agency but require support. We argue that when youth are mobilized in metalevel decision‐making spaces, their engagement is stratified and unequal. We situated youth engagement in decision‐making through the perspective of environmental organizations as a contribution to environmental governance and youth literature.

## Abstract (es)

Participación juvenil en la gestión de la conservación mundial

**Resumen:**

Cada vez reconocemos más el papel importante que desempeñan los jóvenes en la toma de decisiones ambientales relacionadas con la conservación. Lamentablemente, los jóvenes que toman decisiones cruciales suelen quedar excluidos de los espacios de gestión ambiental debido a barreras estructurales, tanto económicas como políticas. La literatura reciente sobre justicia ambiental resalta que esta marginación obstaculiza la participación en el proceso de toma de decisiones. La reciente publicación de la Estrategia para la Juventud 2022‐2030 de la Unión Internacional para la Conservación de la Naturaleza (UICN) ha introducido en el debate a organizaciones ambientales destacadas. El Congreso Mundial de la Naturaleza (CMN) de la UICN constituye un ejemplo útil para comprender cómo los jóvenes acceden y participan en la toma de decisiones al más alto nivel de gestión en una destacada organización mundial de conservación. Utilizamos métodos de etnografía de eventos y observación a los participantes para estudiar el Foro del CMN en Marsella, Francia (2021). Tratamos de examinar las complejidades geopolíticas del poder y las desigualdades subyacentes en la raíz de la participación de los jóvenes, o la falta de ella. Consideramos el compromiso de la UICN con la juventud y esbozamos el proceso desde resoluciones y recomendaciones anteriores hasta la publicación de la Estrategia de la Juventud de la UICN en 2022. Los resultados de las narrativas de los jóvenes que recopilamos mostraron que los jóvenes no son un monolito, que el simbolismo debe ser desafiado, y que los jóvenes tienen agencia, pero requieren apoyo. Sostenemos que cuando se moviliza a los jóvenes en los espacios de toma de decisiones a meta nivel, su participación está estratificada y es desigual. Situamos la participación de los jóvenes en la toma de decisiones desde la perspectiva de las organizaciones ecologistas como una contribución a la literatura sobre gobernanza medioambiental y juventud. © 2024 by John Wiley & Sons, Inc.

## 青年参与全球保护治理

【摘要】 越来越多的人认识到, 青年在围绕保护的环境决策中发挥着重要作用。遗憾的是, 由于经济和政治等结构性障碍, 作为重要决策者的青年往往被排除在环境治理之外。正如最近的环境正义文献所强调的, 这种边缘化阻碍了其积极参与决策过程。最近, 世界自然保护联盟(IUCN)发布的《2022–2030年青年战略》让著名环保组织也参与到这场辩论中来。IUCN世界自然保护大会提供了一个有用的范例, 让我们了解青年如何进入并参与这一重要的全球自然保护组织中最高管理层的决策。我们采用事件民族志和参与观察法研究了2021年在法国马赛举行的世界自然保护大会论坛, 以分析地缘政治中的复杂权力, 以及青年参与与否背后的不平等。我们考虑了IUCN与青年的关系, 概述了从以前的决议和建议到2022年《IUCN青年战略》发布的过程。我们所收集的青年叙述结果表明, 青年并不是不愿改变的铁板一块、象征主义应该受到挑战, 以及青年有能动性但需要支持。我们认为, 在鼓励青年参与元决策空间时, 他们的参与是分层级且不平等的。本研究从环保组织的视角审视了青年参与决策的情况, 希望有助于对环境治理和青年文献。**【翻译:胡怡思;审校:聂永刚】**

## INTRODUCTION

Growing environmental concerns, including climate change and biodiversity loss, have placed importance on multiple‐actor solutions that have youth at the forefront of growing global environmental activism. The most prominent example is the climate change movement associated with protests, such as Fridays for the Future spearheaded by Greta Thunberg from Sweden. The active involvement of youth in environmental activism is visible and has reverberated around the world (Han & Ahn, 2020; Thew et al., 2021; Yona et al., 2020). However, similar representations of youth leadership appear to be absent in conservation. To fill this gap, IUCN (International Union for Conservation of Nature) recently launched the IUCN Youth Strategy 2022–2030 for “meaningful youth engagement …to join efforts in reversing biodiversity loss, conserving nature and managing natural resources” (IUCN, 2022a, p. II). Leading up to this strategy, youth engagement was a recurring theme across different platforms at IUCN's World Conservation Congress (WCC) in September 2021. Notably, at the Opening Ceremony, Hollywood actor Harrison Ford spoke about the inclusion of youth in decision‐making:

Reinforcements are on the way; [referring to youth] they are sitting in lecture halls now, venturing into the field for the very first time, writing theses; they're leading marches, organizing communities; they're learning to turn passion into progress, potential into power; but they're not here yet. In a few years they will be here, in rooms like this, and the world will be better off for it…

Ironically, youth, although present at the IUCN Congress, were not in the room with Harrison Ford and other so‐considered important people. Ford's quote illustrates 2 points typical of youth in conservation. First, youth who should be engaging in processes of environmental decision‐making are not visible in high‐level decision‐making and policy‐influencing spaces. Second, youth are simultaneously seen as future problem solvers who will achieve sustainability and address climate change and biodiversity loss (Walker, 2016).

We examined the processes in which youth are actively engaged in environmental decision‐making in conservation. Similar to other studies (Thew et al., 2022, 2011) of youth participation at the United Nations Framework Convention on Climate Change Conference (UNFCCC), we focused on the IUCN WCC Forum held in Marseille, France, in 2021. Our work followed Intergens’ (Youth and Intergenerational Research, Impact and Action group) evaluation of the Intergenerational Partnership for Sustainability (IPS), a review of IUCN's internal youth engagement processes, and the publication of the IUCN Youth Strategy 2022–2030. We situated youth engagement within the global environmental governance (GEG) literature and focused on how youth are described as marginalized and vulnerable in the environmental justice literature yet are mobilized in the governance literature to promote sustainability and create solutions to existing environmental problems.

### Background

To address climate change, global environmental organizations have gradually mainstreamed youth participation into decision‐making processes, for example, the Convention on Biological Diversity (CBD)—COP (Conference of Parties) 15 Youth Summit in 2022, the UNFCCC YOUNGO (youth NGO) international network, and the United Nations Development Program Youth Global Program for Sustainable Development and Peace. This movement toward youth inclusion was spurred by the recognition that youth must be empowered, beyond tokenism, to lead climate action (Nrkumah, 2021; UNDP, 2022). Although climate change issues are important to assess and understand, youth engagement in GEG, biodiversity protection, and conservation, though equally important, has not received the same level of research or interest. Although prominent youth actors, such as Greta Thunberg, are associated with climate change activism, the youth actors speaking out in conservation and biodiversity loss remain less known. For example, Indigenous youth activists are arguably marginalized by existing colonial power structures (Grosse & Mark, 2020). For example, Josefa Cariño Tauli, a young environmental activist of the Ibaloi‐Kankanaey Igorot people in the Philippines and policy co‐coordinator of the Global Youth Biodiversity Network (GYBN), stated at the CBD COP 15, 2021, “I speak to you today as young person…in a space that makes big decisions about our future but remains out of reach for…people who are most affected.” Despite years of environmental activism, Indigenous youth conservation activists do not receive equal media coverage (Grosse & Mark, 2020, p. 148). Similarly, scholars of youth studies and environmental governance have largely focused on youth from the Global North engaging with nongovernmental organizations (NGOs) about climate change (Han & Ahn, 2020; Ojala, 2011; Reimer et al., 2014; Thew et al., 2021; Threadgold, 2011; Yona et al., 2020).

Conservation requires coordination and agreement between a variety of actors and stakeholders to achieve environmental sustainability (MacDonald, 2010). Despite recognizing the importance of youth involvement in conservation, they are traditionally underrepresented in decision‐making (Narksompong & Limjirkan, 2015). Zurba and Trimble (2014) argue a gap exists between youth and natural resource governance because “resource management is inter‐generationally blind” (p. 79). The absence of youth involvement in decision‐making has been labeled an environmental justice issue (Mkhize et al., 2022; van der Westhuizen, 2021) relative to environmental degradation, structural inequalities, resource access, and well‐being (van der Westhuizen, 2021). The World Health Organization considers youth at risk of marginalization because they are less likely to participate in governance and decision‐making due to economic, political, and procedural barriers (WHO, 2019 in Offerdahl et al., 2014; van der Westhuizen, 2021).

Youth engagement involves active and sustained participation in community activities, extending beyond individual involvement to encompass areas that have had low youth engagement historically, such as decision‐making, sports, and schools (Reimer et al., 2014; Rose‐Krasnor, 2009; UNDP, 2022). Engagement is a multidimensional concept that goes beyond the behavioral dimension of participation to emotional responses, knowledge, and behaviors associated with participation (Rose‐Krasnor, 2009).

Zurba et al. (2020) argue that intergenerational partnership in substantive decision‐making has not materialized because youth remain marginalized in GEG. Labeling youth as important actors in decision‐making is crucial because their exclusion in policy processes can have long‐term consequences for conservation sustainability (Reimer et al., 2014). Yet, as Ford said, youth are still in the “classroom” and “are not here yet” at the decision‐making table. The political framing of youth as future leaders rather than current contributors further shapes policy from “defining youth as a potential asset, to a potential societal problem,” depending on their present and future needs (Hart, 2008; Zeldin, 2004, p. 75). The challenge is that youth have become politicized, so their participation is often a token used to suit political interests (Ansell et al., 2012). This highlights the asymmetrical power relations and raises the question: How have GEG NGOs involved young people in their environmental decision‐making processes?

### Literature overview

To examine how youth as key contributors are positioned in governance and disproportionately affected by environmental issues (Reimer et al., 2014), we present an overview of the literature on GEG and environmental justice.

Lemos and Agrawal (2006, p. 298) define environmental governance as a “set of regulatory processes, mechanisms and organizations through which political actors influence environmental actions and outcomes.” Environmental governance is enacted through environmental NGOs, international accords, national policies and legislation, and (but not limited to) transnational institutions (Lemos & Agrawal, 2006). Environmental NGOs have the decision‐making power to influence how natural resources are distributed, consumed, protected, and accessed (Duffy, 2014). At the global scale, governance is considered the interplay of formal and informal arrangements among multiple actors (Duffy, 2014). In the international political economy, resource governance is described as a neoliberal project aimed at restructuring global politics by asking, “what is to be governed (and what is not), who governs and who is governed, how do they govern, on whose behalf, and with what implications” (Duffy, 2013, p. 224).

GEG considers underlying interests and ideas that influence how public and private actors, such as NGOs and organized social groups, engage in conservation governance and practice at the metalevel (MacDonald, 2010). Organized social groups may promote ideological perspectives, elaborated through coordinated processes emerging from historical contexts (MacDonald, 2010). These ideals may be implemented through NGOs and nonbinding instruments, such as the 30×30 Global Biodiversity Framework (GBF), which advocates for setting aside 30% of land to conserve biodiversity by 2030 (Buscher et al., 2017; Butler, 2023; Walker, 2016).

Decision‐making is part of the performative process of policy formation and implementation (ESCAP, 2009). It operationalizes governance through deliberation processes (Allan & Hadden, 2017; Kwon, 2019). NGOs are deliberative actors who use power to persuade other actors to adopt the interests of the NGO through framing and meaning making (Allan & Hadden, 2017; Kwon, 2019). This process is rooted in the NGO's formal (e.g., WCC Member's Assembly) and informal structures (e.g., WCC Forum and Global Youth Summit [GYS]) to make and implement decisions (ESCAP, 2009). These interactions expose the asymmetries in power between actors that exist in these decision‐making spaces. The underlying formal and informal structures that help select participating actors may exclude some groups (Song et al., 2013). The NGOs already possess global legitimacy in conservation processes (e.g., IUCN Red List of Threatened Species). It is the coordinated agreement of relevant actors, stakeholders, or partners that underpins the framing and meaning‐making of these environmental standards, which are then implemented from the global policy‐regulatory level to the grassroots (MacDonald, 2010). As a global NGO, IUCN facilitates discussions and debates among a wide range of environmental stakeholders and actors at the global level at the quadrennial WCC. Depending on the interests at stake and the decision to be made, it can therefore position certain actors or groups in the decision‐making processes.

Environmental justice places youth at the crux of environmental vulnerability due to the unjust burden of responsibility for past and current environmental problems that will affect their future (Mkhize et al., 2022). It calls on youth to be active stakeholders while highlighting the challenges they face in being positioned as marginalized and vulnerable. The environmental justice literature highlights the power dynamics of decision‐making, particularly in relation to marginalized actors. The position of youth can be evaluated through discussions of “the unequal distribution of environmental costs, benefits and associated welfare outcomes…” and “the proximate and underlying drivers of inequalities” (Martin et al., 2020, p. 20). For instance, youth (e.g., Indigenous peoples and women) are disproportionately affected by environmental problems because “…future generations will be even more exposed to the consequences of irresponsible attitudes and behaviors towards nature” (van der Westhuizen, 2021, p. 2). Therefore, within environmental justice, youth who remain marginalized in GEG need partners to establish environmental solutions (Zurba & Trimble, 2014).

Within environmental justice, recognition and participation intersect, whereby recognition, an important step in gaining access to decision‐making processes, addresses diverse identities, cultures, and knowledges (Thew et al., 2021). According to Thew et al. (2021, participation increases recognition and involves the active contribution of actors. Youth actors should therefore be actively involved in decision‐making processes so their ideas and voices are heard and considered. However, youth recognition is incumbent on whether they are classified as vulnerable and in need of empowerment or whether they are viewed as change agents in organizations and spaces of power (Ojala, 2011; Orsini, 2022). Youth studies and environmental justice literature perpetuate this narrative by oscillating the classification of youth from strong, mobilized actors to weak, passive recipients of aid or empowerment programs (Ojala, 2011; Orsini, 2022; Zurba & Trimble, 2014). This in turn influences their positionality. The empowerment narrative sees youth as beneficiaries who need to be educated and trained, rather than simultaneously partnering with them as independent political actors with agency (Orsini, 2022). Engagement in this regard is based on the agency of active participation in public life and, second, on the way in which state and nonstate actors (in this case, NGOs) actively involve youth in their governance structures (Adler & Goggin, 2005; Reimer et al., 2014). However, this varies according to the geopolitical context. The majority of the studies focused on examples from the Global North (Han & Ahn, 2020; Reimer et al., 2014; Thew et al., 2021; Threadgold, 2011; Yona et al., 2020).

With regard to global climate policy, Fridays for the Future mobilized youth to demand immediate reductions in greenhouse gas emissions as a matter of urgency for current and future generations (Han & Ahn, 2020). Although the literature often cites the example of Greta Thunberg, it is crucial to recognize that there are many other youth perspectives that have made significant contributions to the climate movement (Grosse & Mark, 2020; Han & Ahn, 2020; Orsini, 2022; Thew et al., 2020, 2021; Yona et al., 2020). This reliance in the literature on Fridays for Future as an example of youth engagement speaks to environmental justice and interactive governance, raising the question, Which youth voices are positioned to be recognized and heard (Song et al., 2013)? This question is important because the allocation of resources, including funding and media attention, are based on the dominant narratives produced by actors with access to spaces of power. Furthermore, sociopolitical constructions of youth that situate them as subjects rather than political actors are then reproduced in conferences and conventions, where youth can be disconnected from their agency, which affects engagement (Orsini, 2022, p. 4). These narratives are then amplified in policy formation and implementation and the literature.

Therefore, promoting youth engagement in spaces of power situates them as legitimate, essential actors (Thew et al., 2021). For example, the Rio Conference of 1992 emphasized the need to focus on youth to combat the climate crisis. The 2003 World Parks Congress‐Durban called on youth engagement with biodiversity conservation but did not consistently document their roles. As such, engagement in the sphere of conservation necessitates mobilizing youth. In April 2021, IUCN held the inaugural GYS, followed by another GYS at the WCC in 2021 that attracted youth activists, practitioners, and organizations. At both global events, youth were called to voice their experiences and provide solutions as part of the “One Nature, One Future” approach. We considered the experiences of youth and how they navigated the decision‐making spaces of the WCC Forum because we believe these are key factors in conceptualizing youth engagement with global NGOs.

### Situating IUCN in GEG

Established in 1948, IUCN is described as the world's largest conservation organization with over 1400‐member organizations from over 160 countries (IUCN, 2023; Zurba et al., 2020). It is a membership‐based union of government, civil society organizations, and Indigenous people's groups that unite to advance sustainable development and focus on IUCN's mission (IUCN, 2023). It is the “global authority on the status of the natural world and the measures needed to safeguard it” and has the world's most diverse environmental network (UNEP, 2023). The IUCN has the observer status at the UN General Assembly enabling it to “deliver the policy perspectives of its Members at the highest internal level of diplomacy” (IUCN, 2018, p. 3). The IUCN is composed of the WCC, secretariat, council, national, and regional committees and members, and 8 commissions (IUCN, 2022b). Along with the council, the WCC is considered the highest organ of the IUCN and participates in decision‐making along with its members (IUCN, 2022b).

The IUCN is a neutral space that seeks to influence the actions of state and nonstate actors (e.g., businesses, scientists, and Indigenous peoples) by providing information and advice and building partnerships, rather than mobilizing the public to support conservation (IUCN, 2023). Member organizations are part of the democratic process of discussing and approving resolutions and recommendations at the WCC Member's Assembly, which may influence the global conservation agenda (UNEP, 2023). This includes authoritative reports, standards, and guidelines that inform and support global policy, such as the 2030 Agenda for Sustainable Development. Resolutions are aimed at IUCN, and recommendations are directed toward other agencies and a global audience (IUCN, 2018). Such multilateral agreements influence the governance of conservation from global policies to national regulations and laws that influence terrestrial and aquatic conservation efforts. As the largest global conservation NGO, IUCN is therefore a dominant and crucial global actor with the power to influence conservation engagement across sectors, regions, and demographics.

The WCC is envisioned by IUCN as a center for the promotion of broad‐based participation in environmental governance (Adeyeye et al., 2019; Fletcher, 2014). It aims to foster a space where a variety of actors from different backgrounds assemble to deliberate on the responsibilities and the benefits of environmental protection (Adeyeye et al., 2019; Fletcher, 2014). The WCC has 2 components: the Forum and the Member's Assembly. The Forum includes high‐level panels, debates, training workshops, interactive sessions, and press releases that are accessible to all WCC participants (Adeyeye et al., 2019). Conversely, the Member's Assembly “is the governance body of IUCN” that permits only IUCN members to deliberate and vote on strategies for the next quadrennial (it can be observed by nonmembers) (Adeyeye et al., 2019). We did not focus on decision‐making of the Members Assembly, but rather on how youth engaged in the Forum, which has an influence on policy processes.

Decisions that have influenced how IUCN engages with youth were made previously at WCCs, where strategies, such as IPS, were adopted (Resolution WCC‐2008‐Res‐098). These resolutions evolved to the IUCN Youth Strategy launched in September of 2022. We used a qualitative methodological approach to follow these advancements.

## METHODS

**FIGURE 1:**
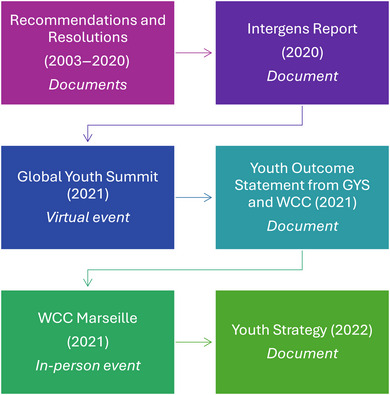
Documents and events that informed the development of the International Union for Conservation of Nature Youth Strategy (IPS, Intergenerational Partnership for Sustainability; WCC, World Conservation Congress).

We chose the IUCN as a case study because it is a global conservation NGO that includes youth in its global events and its organizational framework (IUCN, 2022a). We aimed to determine how IUCN engaged with youth at the 2021 WCC. This research is an independent study and was not conducted in partnership with IUCN, although they were made aware of it and solicited for and participated in interviews. Qualitative design methods were used to collect and analyze data from multiple sources from January 2020 to September 2022. Primary data were collected in 32 semistructured interviews, event ethnography, and participant observation at the WCC in Marseille, France (Brosius & Campbell, 2010; Dumoulin, 2021). Interview questions are in Table 4. Our research protocol followed ethical considerations that prioritize the protection of research participants (Arifin, 2018). Ethical considerations include anonymizing respondents. Secondary data were collected from IUCN reports, documents, and publications (a comprehensive list of the primary and secondary data is in Table 1). Research was conducted from January 2020 with youth from 18 to 35 years old until the publication of the IUCN Youth Strategy 2022–2030. Data were collected following the progression of youth engagement in IUCN as illustrated in Figure 1. S.S. attended the GYS in April 2021 (virtually) and the WCC in September 2021 (in person), and G.W. attended the WCC.

**TABLE 1:** A comprehensive list of the primary and secondary data collected and analyzed from January 2020 to June 2022 in a study of how youth participants are engaging with conservation governance at the International Union for Conservation of Nature (IUCN) World Conservation Congress Forum.

Data category	Data collection method	Duration	Data sample	Data summary	Themes for analysis
Primary data	Semistructured interviews	April 2021 to June 2022	7 internal actors (IUCN Commission members and staff) 21 external actors (composed of activists, practitioners, students, and volunteers) Purposive sampling methods and snow‐ball sampling Youth from countries in the Global South were sought, particularly African youth on the virtual platform of the Global Youth Summit and in‐person at the World Conservation Congress Forum	Internal process of youth engagement Intergens report and the youth strategy Youth expectations of IUCN GYS and WCC Work or activities youth participants are involved in (experiences at the GYS and WCC; conservation topics and issues they wanted to discuss or highlight during sessions)	Inequalities within environmental solutions Future of conservation with youth‐based solutions Transgenerational and intergenerational dialogue Post‐2020 Biodiversity framework = transformative change Youth positionality in solutions
	Event ethnography and participant observation	April 2021 (virtually)	IUCN GYS: 24 virtual events	Formulating the GYS Outcome Statement Dominance of Global North in hosting sessions (Canada, the United States, Japan, China) Climate change and youth engagement Youth and rights to nature and policy	Making space for youth voices. Mobilizing and rallying youth for biodiversity protection Education and storytelling
		September 2021 Marseille, France (in‐person)	IUCN WCC: 10 in‐person sessions	Reactions from the GYS Outcome Statement Transgenerational approaches to conservation rights and duties	Intergenerational dialogue Private sector engagement
Secondary data	Document analysis	January 2020 to September 2022	Press releases: #NatureforAll youth champions (IUCN, 2021) “One Nature, One Future: Together we can!” (IUCN, 2021)	Youth definitions and classification Youth engagement objectives Strategies to establish youth engagement Responsibility of youth Social and environmental movement objective for youth‐in‐action Youth roles Youth incentives for engagement	Power dynamics between organizations versus youth Youth definition and classification importance Youth engagement strategy evolution for IUCN
		2021–2022	IUCN reports: IUCN Youth Strategy 2020–2030 (IUCN, 2022) #NatureForAll Youth champions Annex I and II (IUCN, 2021)	Youth definitions and classification Decision‐making inclusion of youth Youth engagement objectives Position of IUCN with youth Responsibility of youth	Tracing youth incentives for engagement Narratives of youth engagement held by global NGO
		2003–2021	Resolutions and recommendations from 2003 World Park's Congress and 2004–2021 WCC	Younger generations gaining empowerment in relation to protected areas, which involves giving them the tools and resources to participate in conservation efforts and make a positive impact on the environment IUCN focused on capacity building for young professionals, aiming to equip them with the necessary skills and knowledge to contribute effectively to conservation and environmental initiatives Intergenerational partnerships fostered to promote ethical leadership, ensuring a just, sustainable, and peaceful world for present and future generations Efforts made to increase youth engagement and promote intergenerational collaboration across the IUCN, encouraging young people's involvement in environmental issues Goal is to connect people with nature on a global scale, emphasizing the importance of appreciating and preserving the natural world Children and youth play a crucial role in conservation, recognizing their potential as agents of positive change and key stakeholders in environmental decision‐making processes Creation of an ombudsperson for future generations being considered, which would be an independent authority responsible for protecting the rights and interests of future generations, ensuring sustainability and responsible decision‐making	
		September 2021	GYS Outcome Statement (IUCN, 2021)	Resolutions to engage youth Position of youth at IUCN GYS and WCC IUCN response to Intergens report	
		January 2019 and January 2021	Other reports: ‐ Canadian Wildlife Federation: Youth engagement at IUCN WCC. ‐ Summary Report, UNIL Delegation at the IUCN WCC in Marseille.	Intergenerational partnership strategies in IUCN Mechanisms to include youth youth definitions and classification	

Abbreviations: GYS, Global Youth Summit; IPS, Intergenerational Partnership for Sustainability; UNIL, University of Lausanne; WCC, World Conservation Congress.

Conferences and events are described as coconstructed environments in which event‐centred methodological challenges are raised (Schulte‐Romer & Gesing, 2023). These spaces offer an opportunity for researchers to meet and mingle with key stakeholders from various backgrounds (Schulte‐Romer & Gesing, 2023). S.S. was a member of the initial steering committee for the IUCN GYS from October 2019 to January 2020 and volunteered as a session rapporteur at the WCC, documenting session themes, notable statements, and impactful quotes or questions from the panel and audience. Data analysis included content and thematic analysis (Stemler, 2015). Primary and secondary data were organized and coded in Atlas TI to categorize sources and identify patterns and relationships among sources.

## RESULTS

### Situating youth engagement at the WCC

IUCN has endeavored to engage with youth for nearly 20 years through the WCC Resolutions and Recommendations (2003–2022) (Table 2). Their youth engagement model for decision‐making is currently and primarily focused on youth who are members of IUCN commissions or working in the secretariat, even though during the period of research the “One Nature, One Future: Together We Can!” call to action affirmed IUCN's commitment to engage with all youth.

**TABLE 2:** List of World Conservation Congress (WCC) resolutions related to youth or youth engagement from 2003 to 2021.

Year	Location	Resolution code	Description
2003	Durban, South Africa World Parks Congress	Outcome 6 (Durban Action Plan)	Younger generations are empowered in relation to protected areas
2004	Bangkok, Thailand	WCC‐2004‐Res‐029	Capacity building of young professionals within the union (IUCN, 2004).
2008	Barcelona, Spain	WCC‐2008‐Res‐098	Intergenerational partnerships fostering ethical leadership for a just, sustainable, and peaceful world (IUCN, 2008).
2012	Jeju, Korea	WCC‐2012‐Res‐008	Increasing youth engagement and intergenerational partnership across and through the union (IUCN, 2012).
2016	Hawaii, USA	WCC‐2016‐Res‐085	Connecting people with nature globally (IUCN, 2016).
2020	Marseille, France	WCC‐2020‐Res‐062 WCC‐2020‐Res‐046	Role of children and youth in conservation Creation of ombudsperson for future generations

Prior to the GYS and the WCC, the IPS Review, written by the Intergens group (which includes several members of the IPS), identified the call for youth engagement at the 5th IUCN World Parks Congress in Durban, South Africa (2003), as a defining moment (Zurba et al., 2020). This call encouraged young commission members to achieve intergenerational connections across IUCN and gave official recognition to young leaders (Zurba et al., 2020).

The Intergens report assessed the feasibility of implementing youth engagement in a large NGO. It questioned the lack of youth in IUCN's existing internal governance and decision‐making framework. As such, it proposed IPS as a necessary tool to include youth through intergenerational dialogue, collaboration, and knowledge sharing (Zurba et al., 2021). Furthermore, it recommended a Youth Endowment Fund for “sustained financial backing for the implementation of youth engagement and the IPS mandate” from IUCN (IUCN, 2021, p. 4). This was denied by IUCN leadership and opened to its partners for consideration (IUCN, 2021).

The Intergens report highlighted the structural challenges associated with the prolonged democratic processes and consultations of a large NGO. For example, recommendations were not implemented in their entirety because it was not explicitly isolating youth as individual actors within IUCN (internal participant 1). It required youth to engage with the guidance or mentorship of an “adult” internal member or employee through the “buddy” system (internal participant 1) (Zurba et al., 2020). The complexity stemmed from differentiating between those in the youth bracket (as mentees) versus those outside it: “How do you tell someone they are not a youth?” (internal participant 1). As a result, the Heritage, Culture and Youth team was established within the IUCN Secretariat in 2021, and a separate youth strategy was established (internal participant 1). The components of IPS were thus merged with the first IUCN GYS and in the development of the IUCN Youth Strategy.

The youth strategy was established by youth within IUCN in consultation with youth‐led organizations (e.g., GBYN, YOUNGO, UNEP) and contained suggestions from the Outcome Statement of the GYS (April 2021) to ensure they reflected the broad perspectives of youth in conservation (IUCN, 2022a). Finalized in May 2022, the strategy responded to the 3 themes we identified and discuss below. It established ways for youth to engage with IUCN's decision‐making structures. These infrastructures included the Youth Advisory Committee (which incorporates the commissions and secretariat) and increased job prospects (IUCN, 2022a, p. 5). Its aims to entrench youth “perspectives, inclusion and empowerment in all parts and all levels of the Union” through intergenerational collaboration (IUCN, 2022a, p. 1). Using age as the key determinant, the strategy refers to “youth” aged between 15 and 24 and “young professionals” aged between 18 and 35 (IUCN, 2022a). Its guiding principles aim to increase diverse voices and perspectives (e.g., gender, race, context, and disabilities). It positions youth as leaders who can champion initiatives and influence decision‐making processes to overcome tokenism. However, the Action Framework and specific short‐term priority actions only facilitate effective and sustained youth participation and cross‐generational collaboration at all levels within IUCN (IUCN, 2022a). Although the GYS and the WCC opened engagement to youth external to IUCN, youth activists and practitioners were concerned that IUCN is negating the environmental justice issues youth face in their daily experiences.

### Challenge tokenism

The following experiences of youth at the WCC Forum are presented relative to the 3 themes that emerged from youth participant narratives at the WCC: challenge tokenism, youth are not a monolith, and youth have agency but require support. The WCC had a myriad of actors (e.g., economic and political executives and environmental practitioners). In light of the COVID‐19 pandemic, the WCC Manifesto promoted “One Nature, One Future” by committing to the post‐2020 biodiversity goals: mitigating climate change, supporting the 30×30 GBF, and encouraging stronger partnerships among stakeholders, especially youth. Key events included the official opening ceremony and the on‐site GYS.

The GYS saw youth activists and leaders discussing environmental issues and solutions. A product of GYS was the Outcome Statement that showed how youth have been mobilized globally. It highlighted youth agency in policy making and challenged tokenism by urging decision makers to actively engage with the youth by providing financial means, capacity building, and digital technologies to create more inclusive spaces for marginalized groups. Furthermore, youth described themselves as capable leaders who are already spearheading innovative initiatives that are tackling climate change and biodiversity loss through community work. The Outcome Statement (IUCN Global Youth Summit, 2021) crystalized the needs of youth by capturing their challenges while advocating for transformative solutions generated from their local contexts. It argued that Indigenous peoples and knowledge can inform solutions to threatened nature and that their experiences (historical and contemporary) need to be at the forefront of decision‐making agendas that affect people who cannot attend such meetings. It showed that an intersectionality of various spheres (social, cultural, economic) in society and age groups is essential for sustainability. This was an important moment for youth engagement in GEG and highlighted a need for more initiatives promoting intergenerational dialogue at both international policy and local level.

By contrast, the WCC was opened by an exclusive panel of world leaders from politics, finance, and other industries (Table 3 ) and excluded youth leaders. It was a visible representation of how influential and important IUCN is in geopolitics and GEG. However, the high‐level panel lacked representation from Indigenous peoples, local community leaders, and youth, the very groups identified in environmental governance and justice literature as marginalized and vulnerable. It was a closed event where only invited guests, delegates, and persons of high status or rank could enter. Harrison Ford's speech aptly spoke to this lack of representation: “… they're not here yet….”

**TABLE 3:** People in attendance at the high‐level panels for the official opening ceremony of the World Conservation Congress on 9 March 2021.

Name	Title or affiliation
Emmanuel Macron	President of the French Republic
Mr. Mahamdou Issoufou	Former President of Niger
Frans Timmermans	Vice President of the European Commission
Christine Lagarde	European Central Bank President
Gilbert Fossoun Houngbo	President of the International Fund for Agricultural Development
Barbara Pompili	French Minister for the Ecological Transition
Sebastiao Salgado	Brazilian social documentary photographer and photojournalist
Kyriakos Mitsotakis	Prime Minister of Greece
Charles Michel	President of the European Council
Harrison Ford	Conservation International and Hollywood actor

**TABLE 4:** List of the key interview questions used during interviews with youth participants within and outside the International Union for Conservation of Nature during and after the Global Youth Summit and World Conservation Congress.

Category	Question
General demographic information	What is your name and how old are you? How would you describe your current role or position (employed, student, entrepreneur)? Would you describe yourself as a youth/young person?
Affiliation with IUCN	How are you affiliated with IUCN? Had you worked with IUCN prior to the World Conservation Congress? Are you an IUCN Commission Member?
Attendance at the World Conservation Congress (WCC) and Global Youth Summit (GYS)	How did you hear about the WCC? Is this your first time attending? Are you attending as a participant or speaker? What has your experience been of the WCC or GYS? Did you contribute to the GYS Outcome Statement after the GYS? Have you been able to meet other youth participants? Did you encounter any challenges to come to France (Marseille)? Did you receive any funding/sponsorship to attend? What sessions have you been attending? What are the highlights and key takeaways?
Youth Engagement in IUCN	How was information captured to formulate the Outcome Statement? Was there an even distribution of attendance of youth from different regions? Which youth groups, movements, and organizations were most prominent during the WCC and GYS? What would “intergenerational” engagement be operationalized on a day‐to‐day basis in the IUCN Secretariat and IUCN commissions? Is implementing the IUCN Youth Strategy a recommendation or requirement for IUCN leadership? How would mainstreaming youth outside of IUCN be implemented?

The youth who participated in the Official Opening Ceremony were featured as dancers. This called into question the purpose of having a separate GYS. The separate organization of these 2 prominent events showed that the proposal for intergenerational dialogue (between youth and world leaders) in the Outcome Statement and the Intergens report was not fully put into practice on the opening day of the WCC Forum. The GYS was arguably siloed, depriving youth of the opportunity for dialogue between world leaders and youth leaders. Princess Laurentien of the Netherlands stated the following at the GYS, “…I'm incredibly restless…I'm not sure who I should be talking to. Should I talk to the young people or the empty tables of the presidents, ministers; where are they?”

Although she is not a youth actor, her question demonstrates that there was a stratified engagement, which could be seen as tokenistic. External participant 6, an important volunteer, noted the absence of Indigenous peoples and youth: “…we know that these people (VIP) are, at least, as effective as any development bank for conserving nature, [but] we may [need to] think about who are the true very important people in conservation's world?”

Hindou O. Ibrahim, an Indigenous youth activist from Chad, stated that youth were overlooked when it came to discussions at the high‐level panel of the opening ceremony and questioned IUCN's target audience at the WCC. She noted (referring to the opening ceremony during her speech at a high‐level session), “…They talk about how important we are, but we are not at the table with them.”

The GYS discussions that preceded the opening ceremony emphasized the importance of youth “investing in their own future” and the need for intergenerational cooperation and dialogue between leaders and decision makers and youth actors (IUCN WCC, 2021). The siloed GYS and opening ceremony contradict the “One Nature, One Future! Together we can!” approach, which called on all WCC participants and partners to work with youth as “leaders of conservation, not victims” (IUCN WCC, 2021). A primary theme of the GYS was “youths’ active engagement” to avoid being dismissed through tokenism by powerful actors. Youth speakers at the GYS pled for global leaders to “act now” to mitigate the effects of continued biodiversity loss and climate change. Brighton Kaoma, a Zambian social entrepreneur, stressed that tokenistic engagement by global leaders is an obstacle to finding sustainable solutions: “Global leaders, we are tired of your reactions and words, we need you to act now! If you can't act, allow the young people to lead!”

The call to act now was a sentiment shared by most speakers and youth participants. The importance of intergenerational dialogue was raised in the Outcome Statement but was lost in the selection of the high‐level panel for the opening ceremony. Youth participants could have contributed by representing the youth experience, as outlined in the Outcome Statement, to the High‐Level Panel and to the international media present.

### Youth are not a monolith

In youth‐centered sessions on governance and activism, panelists actively shared their experiences and concerns. The youth participants found the sessions to be interdisciplinary (including representatives from business, academia, and activism) and provocative. Youth challenged the decision makers present to be more proactive and inclusive—for example, utilizing bottom‐up approaches, such as storytelling, to raise awareness on biodiversity loss. At the panel discussion Youth Voices for Nature and People in 2007, GYBN and the United Nations Educational, Scientific and Cultural Organization (UNESCO) discussed the importance of a unified vision to communicate the common feelings of youth. They argued that honest conversations about the environmental crisis must involve cross‐cutting political, moral, social, and environmental issues. Pravalli Vangeti (UNESCO) said that communication that breaks down the silos of top‐down policy making is key to empowering youth. Through bottom‐up education initiatives that combine traditional knowledge with modern technologies (social media), youth narratives can be amplified.

However, in a panel interview with youth activists, Melina Sakiyama (GBYN‐Brazil) and Nisreen Elsiam (UNESCO‐Sudan) noted that the experience of youth activists in the Global North differs from that of youth activists in the Global South. They highlighted that IUCN's location in the Global North affects the agenda of WCC events. This leads to inequalities, for example, in the protection of environmental activists: “Why does the media focus on some and not others?” Their concern for environmental activists in Sudan or Brazil stems from the “deadly” and “gruesome struggle” such actors face in the fight for justice (environmental defenders are often killed). They explained that European activism is “white collar” and “civilized,” whereas in the Global South “you are defending your life as well as the environment.” Both activists agreed that spaces like the WCC do not discuss these “inconvenient truths.”

External participant 10, a youth activist from Kenya, faces barriers to project funding and access to basic services that hinder her work. In Kenya, she works with young women from rural areas who face food insecurity due to drought and human–wildlife conflict. Similarly, external participant 11 from Zimbabwe stated that the WCC should also reflect the “human aspect” of conservation work, along with policy (cultural sensitivities of rural youth and their experiences). In Zimbabwe, youth are interested in biodiversity conservation in communities bordering national parks, but the youth lack basic resources, such as running water, and are more concerned with “passing their exams” in school. Education makes them aware of animals and human–wildlife conflict, but their basic needs come first. He also expressed his gratitude for the opportunity to travel for the WCC. Youth his age face challenges in securing meaningful employment, which hinders their ability to express their passion for the environment and attend events outside the country. According to external participant 6, “I came here through my organization, and I hope to be able to meet other people to share my experiences with….”

Youth participants from the Global South were aware of the structural barriers that have hindered, and may continue to hinder, their ability to access international events. They pointed out that the WCC is mainly hosted in the Global North and therefore resources are focused on those who can afford to attend. This is problematic because it further entrenches environmental inequalities, especially when it comes to discussing solutions. The IUCN has the power and capacity to host the WCC with prominent figures in attendance. Youth participants expressed concern that the WCC is a privileged environment to make decisions on behalf of youth around the world. The inequalities in access to the WCC were based on location (France) and the cost of registration. The IUCN registration fee alone is a barrier to access. Youth participants (aged 15–24) paid 200 Euros to register, even though their youth, young adult, or young professional categories extended to 35 years of age. This meant that other youth participants over 24 (considered young people) paid either the commission member fee of 780 Euros or the full registration fee of 1200 Euros. The registration fee reflects existing structural inequalities. Like others, external participant 6 participated through his organization, which covered the costs. For many youths in the Global South and North, such fees put attendance out of reach.

Interviewees such as Melina Sakiyama, noted the need to recognize that engagement at the WCC has not been inclusive or meaningful due to the lack of adequate youth representation who are or will be affected by decisions taken at the WCC Assembly. As such, youth are not a monolith and neither are their experiences or their ability to participate in global fora.

### Youth have agency but require support

Our results showed that one of the main barriers to engagement is funding. Participants noted that funding is highly politicized. It is often based on English language skills and the ability to properly synthesize the needs, actions, and outcomes of those applying. This insight emerged from the rural–urban experience. Rural youth may lack access to technology and ability to effectively express their concerns, but their needs matter. Decisions are therefore focused on the experiences of youth (mainly from the Global North) and their ideas and solutions to the exclusion of those who are not part of the GYS and WCC forum. Participants emphasized that their diversity should be recognized by creating spaces of engagement and in designing policies. They pointed out that youth “have a lot to say and are frustrated” and “need safe spaces” to share their experiences and ideas without fear of losing access to future opportunities and jobs.

Melina Sakiyama (GBYN‐Brazil) and Nisreen Elsiam (UNESCO‐Sudan) noted that the work of environmental activists has been severely affected by COVID19. Melina stated that “hope is limitless, but it cannot feed anyone,” as she explained the difficulty of achieving goals at the grassroots level. They noted that a generational gap between those in power and the youth exists due to the “difference in thinking and ideas.” Through their experiences, they noted that youth are expected to have hope, but they suffer from depression and environmental insecurity due to low wages and marginalization. In addition, Melvin Flores from the Global Youth Statements on Nature Based Solutions panel said that different cultures and ethnic groups need their traditional knowledge to be recognized in education systems. He explained that “in Guatemala, 21 Indigenous communities have lived with the forests for many years, and they get medicines… (yet) they are willing to learn from us.” The recognition of Indigenous knowledge and its role in conservation practices that have protected forests and lands for centuries needs to be considered. Therefore, using environmental education, powerful actors should work closely with youth to disseminate information, communicate, and devise standards for awareness raising that reflect traditional knowledge of the local environment.

Our findings showed that IUCN recognized the need for global youth engagement by hosting the GYS virtually (April 2021) and in‐person at the WCC (September 2021). This created opportunities for some youth participants to voice their opinions and concerns for conservation in sessions and events throughout the WCC Forum, but barriers to participation remain.

## DISCUSSION

“One Nature,” the WCC theme, addressed how the world's population shares the planet's biodiversity and finite resources. “One Future” was the call to action for participants to secure the future by working together. The IUCN is a prominent geopolitical actor with the power to set new standards and practices in motion. Practices, such as youth engagement, have been demonstrated by IUCN through implementation of processes in its internal governance structures (commissions and secretariat), creation of spaces for engagement through both the GYS and the WCC Forum (2021), and articulation of youth concerns and expectations in the Outcome Statement and the youth strategy 2020–2030. However, youth engagement in the WCC Forum, a space that influences decision‐making, revealed barriers to youth engagement at the global level.

The environmental justice literature shows complex dynamics that highlight power asymmetries through youth justice claims‐making. Youth justice claims‐making is described as the articulation of youth experiences and needs through the claiming of organizational resources, and in return, the organization (the more powerful actor) either endorses or rejects it (Thew et al., 2020). Under the guise of “One Nature, One Future,” justice claims were made in both GYS because youth actors needed the WCC Assembly and the IUCN Council to endorse the proposals made. Youth engagement in this regard speaks to their position of self‐mobilization and having power to negotiate based on justice claims, to mobilize, and to negotiate for power. This speaks to the subjectivities and positionalities of youth in the GEG. In the conservation arena of the WCC Forum, the youth narratives showed that activist work is being done, albeit in localized clusters at the individual or organizational level. Furthermore, these experiences were voiced in the GYS and through the Outcome Statement.

The GYS Outcome Statement and the Intergens report strongly recommended intergenerational partnerships and dialogue. The global youth voices represented in the Outcome Statement were not shared with the high‐level panel's political and economic leaders. Mobilizing youth from across the world firstly involves NGOs recognizing the importance of including marginalized groups in key governance spaces. The narratives in this study highlighted that youth participants face different challenges to engagement because power dynamics need to be addressed to strengthen intergenerational dialogues and partnerships. Ideas and knowledge structures are based on assigned meanings and interpretations of issues, for example, nature‐based solutions for Indigenous peoples versus policy makers (Taylor, 2000). This social constructionist perspective is rooted in traditional top‐down flows of information (Taylor, 2000). Environmental justice challenges these constructions and encourages dialogue through avenues such as intergenerational knowledge sharing. The official opening ceremony was a prime opportunity to implement IPS on a global platform through IUCN. The experiences of activists promoting justice in countries, such as Brazil, were described as “deadly” and European activism as “civilized.” These diverse youth experiences in biodiversity protection need to be amplified in high‐level spaces to raise awareness.

The WCC strengthens the integration between the private sector and conservation in relation to neoliberal modes of environmental governance (Buscher et al., 2012; Brockington et al., 2008; Fletcher, 2014; MacDonald, 2010). This highlights the asymmetrical power dynamics that exist in governance between actors, with the WCC Forum focusing on political and business interests, as highlighted at the Opening Ceremony (Shackleton et al., 2023). Although participation processes may have the best intentions, they are embedded in power (Carpenter, 2020). For instance, through “shallow participation,” powerful stakeholders may drive processes that foster participation while retaining the decision‐making power (Shackleton et al., 2023, p. 11). This may reinforce existing power dynamics, especially when underlying structural power remains unchecked (Shackleton et al., 2023). For example, although the slogan “One Nature, One Future!” may encourage unity, it does not adequately address the underlying inequalities affecting youth and other marginalized groups in these decision‐making platforms. Youth experiences with conservation are not homogenous. Orsini (2022, p. 29) rightfully states, “there is no ‘global youth’ but a diversity of youth actors in global politics.”

The IUCN's internal youth engagement process aims to adapt governance structures to include youth voices in key components of the Union (e.g., Secretariat and Council). This process was a joint effort among members (voting on resolutions and recommendations), the secretariat (internal survey and discussions), and the council. It also included obtaining input from youth at the GYS (April 2021). This process resulted in an Outcome Statement that informed the youth strategy. However, this output primarily applies to youth within IUCN and does not address engagement in decision‐making for those outside of IUCN. As highlighted above, external youth engagement is based on collaboration and partnership through intergenerational dialogue at events and involvement in meaningful roles (IUCN, 2022a). Although the focus was on unity in experience and action under “One Nature, One Future,” youth engagement at the WCC also highlighted the underlying inequalities that affect who can afford to be at the table.

Youth interactions revealed that IUCN has visible, formalized structures that indirectly act as gatekeepers to engagement and result in unequal access to spaces of power. For youth who face an intersection of socioeconomic challenges in their daily lives (e.g., unemployment), the IUCN access policy, embedded in the cost of registration, is a visible barrier to engagement. Access to the WCC limits participants from the Global South who face physical and material barriers due to high registration costs. Furthermore, the WCC promotes traditional and elite power structures that are embedded in an event landscape fostered in a corporate environment (George & Reed, 2017). Traditional and narrow governance structures are maintained to obtain funding or appease donors (corporate strategies) (George & Reed, 2017). Thus, one youth participant rightly asked, “Who are the real very important people in the world of conservation?” In addition, hosting a separate GYS, where an alternative opening ceremony invited global leaders, ended up siloing youth engagement with a result of youth preaching to the choir and empty tables.

The IPS initiative (Zurba et al., 2021) creates a realistic starting point for international NGOs to recognize and represent youth on an equitable basis, using a sustainable approach. It would be idealistic to expect IUCN to shift its focus from business and state partners to primarily associating with actors such as youth and Indigenous peoples. Instead, through the IPS or an amended version, IUCN can create safe engagement spaces with global leaders, fostering dialogues with youth in order to transform their ideas and solutions into policy. Within this multistakeholder approach of intergenerational dialogue, those with power (in business and politics) can discuss sustainable solutions to context‐specific environmental issues being faced by youth. The IUCN has the geopolitical power to bring together actors in a collaborative platform for dialogue. This can be described as the safe spaces approach to IPS, which would allow youth the opportunity to voice concerns, frustrations, and challenges without fear of repercussions from the leaders present. In the context of environmental justice, youth disproportionately affected by the unequal distribution of environmental costs could expect action‐oriented responses and support that acknowledges and addresses their diverse identities, cultures, and experiences. Orsini (2022) therefore suggests that youth self‐mobilize and express their innovative and cross‐cutting demands (bridging environmental ambitions, accountability, and human rights) should be encouraged at global events such as the WCC. This mobilization will help ensure that youth become even more visible, especially where protecting and conserving biodiversity is concerned, both in the literature and in policy.

Conservation governance has been criticized because of its exclusionary approaches (Brockington & Wilkie, 2015; Buscher & Ramutsindela, 2016; Techera, 2008). This has led to decades of disenfranchisement of marginalized groups, such as local communities and Indigenous peoples and more recently youth. Environmental justice calls for the recognition and inclusion of these marginalized groups in decision‐making processes (Boon, 2010). For youth, engagement depends not only on motivating decision makers to be inclusive but also on creating structural mechanisms to enable participation, where space is created and social recognition is granted (Yohalem & Martin, 2007).

We found that although youth engagement is recognized by global conservation actors, such as IUCN, it is stratified and can be viewed as tokenistic due to the underlying inequalities resulting from access to decision‐making spaces and resources controlled by powerful actors.
